# Author Correction: A nanoluciferase SARS-CoV-2 for rapid neutralization testing and screening of anti-infective drugs for COVID-19

**DOI:** 10.1038/s41467-021-24300-8

**Published:** 2021-06-21

**Authors:** Xuping Xie, Antonio E. Muruato, Xianwen Zhang, Kumari G. Lokugamage, Camila R. Fontes-Garfias, Jing Zou, Jianying Liu, Ping Ren, Mini Balakrishnan, Tomas Cihlar, Chien-Te K. Tseng, Shinji Makino, Vineet D. Menachery, John P. Bilello, Pei-Yong Shi

**Affiliations:** 1 Department of Biochemistry and Molecular Biology, University of Texas Medical Branch, Galveston, TX USA; 2 Department of Microbiology and Immunology, University of Texas Medical Branch, Galveston, TX USA; 3 Department of Pathology, University of Texas Medical Branch, Galveston, TX USA; 4 Gilead Sciences, Inc., Foster City, CA USA; 5 Institute for Human Infections and Immunity, University of Texas Medical Branch, Galveston, TX USA; 6 Sealy Institute for Vaccine Sciences, University of Texas Medical Branch, Galveston, TX USA; 7 Sealy Center for Structural Biology & Molecular Biophysics, University of Texas Medical Branch, Galveston, TX USA

**Keywords:** High-throughput screening, SARS-CoV-2

Correction to: *Nature Communications* 10.1038/s41467-020-19055-7, published online 15 October 2020

The original version of this Article contained an error in the Acknowledgements, which incorrectly listed the NIH grant ‘AI142759’. This has been corrected in both the PDF and HTML versions of the Article.

